# Analysis of the relationship between mathematical learning stress and neuroendocrine response based on causal modeling

**DOI:** 10.3389/fendo.2026.1886711

**Published:** 2026-09-22

**Authors:** Lei Shi

**Affiliations:** School of Mathematics and Physics, Hulunbuir University, Hulunbuir, China

**Keywords:** causal modeling, mathematical learning stress, neuroendocrine responses, optimal transport, Representation-Factorized-Optimal

## Abstract

**Introduction:**

This study develops a methodological framework to investigate the relationship between mathematical learning stress and neuroendocrine responses through causal modeling. Traditional approaches often fail to capture the complex interplay between psychological stressors and physiological responses, limiting their ability to provide actionable insights. To address these limitations, we propose the Representation-Factorized-Optimal (RFO) model, which is specifically designed to model the intricate dynamics between stress experienced during mathematical learning and the associated neuroendocrine responses.

**Methods:**

The model integrates three core components: the Representation Consistency Enforcer (RCE), which ensures the stability and reliability of variable representations; the Factorized Relation Projector (FRP), which disentangles and projects the relationships between stress and neuroendocrine variables; and the Optimal Transport Matcher (OTM), which aligns these variables for robust causal inference. To further enhance the model’s performance, we incorporate advanced techniques such as representation alignment and sparse representation learning, enabling the extraction of meaningful patterns from high-dimensional data.

**Results and Discussion:**

Experimental results demonstrate that the proposed model achieves superior accuracy and interpretability compared to existing methods, providing a more nuanced understanding of the causal mechanisms underlying the interaction between mathematical learning stress and neuroendocrine responses. These findings hold significant implications for advancing research in the interdisciplinary fields of educational psychology and neurobiology, offering a robust tool for exploring the physiological impacts of cognitive stressors.

## 1 Introduction

In recent years, the relationship between mathematical learning stress and neuroendocrine response has garnered significant attention due to its implications for educational practices and mental health. Understanding this relationship is not only crucial for developing effective educational strategies but also for addressing the broader impacts of stress on cognitive and physiological health Degtyareva et al. (1). The necessity of this research is underscored by the increasing prevalence of stress-related disorders among students, which not only affects their academic performance but also their overall well-being Stroud et al. (2). Moreover, exploring this relationship through causal modeling offers a robust framework for identifying underlying mechanisms and potential interventions Rotar (3). This approach not only enhances our comprehension of the stress-response system but also provides a foundation for developing targeted strategies to mitigate the adverse effects of stress in educational settings Chung et al. (4).

Early efforts to model the relationship between stress and neuroendocrine responses relied on structured frameworks that utilized predefined rules and expert-driven insights. These approaches aimed to capture the dynamics of stress responses by explicitly encoding domain-specific knowledge into computational models Ursino and Cristalli (5). While these methods provided interpretability and consistency, they were constrained by their inability to account for the variability and complexity inherent in human stress responses Laketic et al. (6). Their rigid structures often struggled to adapt to novel scenarios or incorporate new data, limiting their applicability in diverse educational contexts Kassymova et al. (7).

To address these limitations, researchers began employing statistical models capable of identifying patterns and dependencies within structured datasets. Techniques such as regression analysis, decision trees, and clustering algorithms offered a more adaptable framework for understanding the interplay between stress and neuroendocrine activity Ogakwu et al. (8). These methods demonstrated improved flexibility and predictive accuracy, enabling the analysis of more complex interactions Tang et al. (9). However, their reliance on manually crafted features and the need for extensive domain expertise posed significant challenges, particularly in capturing the multifaceted nature of stress responses Ramirez et al. (10).

The introduction of advanced neural network architectures has transformed the field by enabling end-to-end learning from raw data. Deep learning models, such as convolutional and recurrent neural networks, have shown exceptional capability in uncovering intricate patterns and hierarchical relationships within large datasets Beilock (11). Transfer learning techniques, utilizing pre-trained models, have further enhanced the generalizability of these approaches, allowing for effective application even in scenarios with limited data availability Rattanakul and Lenbury (12). Despite their remarkable performance, these models often face challenges related to computational demands and interpretability, underscoring the need for balanced solutions that combine accuracy with transparency in educational and physiological research Rodríguez-Arce et al. (13).

Based on the aforementioned limitations, we propose a novel approach that integrates causal modeling with advanced machine learning techniques to better understand the relationship between mathematical learning stress and neuroendocrine response. Our method addresses the interpretability issues of deep learning models while maintaining their predictive power, offering a more comprehensive and practical solution for educational and physiological research. By combining causal inference with machine learning, we aim to provide a more nuanced understanding of the stress-response system, enabling the development of targeted interventions to improve student well-being and academic performance. For readability and consistency, we refer to the proposed Representation-Factorized-Optimal model as RFO throughout the remainder of this manuscript. Similarly, the three major modules are abbreviated as RCE for the Representation Consistency Enforcer, FRP for the Factorized Relation Projector, and OTM for the Optimal Transport Matcher.

We summarize our contributions as follows:

- Our approach enhances model interpretability by integrating causal inference with machine learning, providing clearer insights into the stress-response relationship.
- The method demonstrates high efficiency and adaptability across various educational and physiological scenarios, ensuring broad applicability.
- Experimental results show significant improvements in predictive accuracy and robustness, validating the effectiveness of our approach in real-world settings.

## 2 Related work

### 2.1 Mathematical learning stress impact

The influence of stress on mathematical learning has been extensively examined, with particular attention to its effects on cognitive processes and academic performance Ramirez et al. (10). Stress, characterized as a physiological and psychological reaction to perceived challenges, has been shown to significantly alter the ability to engage in mathematical reasoning Ogakwu et al. (8). Studies have demonstrated that stress impairs critical cognitive functions such as working memory and attention, which are essential for solving mathematical problems Vogel and Schwabe (14). In learning contexts, stress can influence not only immediate task performance but also memory encoding, consolidation, and retrieval, thereby affecting how students acquire and retain mathematical knowledge. This connection is particularly important because mathematical learning often requires the coordinated use of attention control, working memory, and long-term memory retrieval under time constraints. Activation of the hypothalamic-pituitary-adrenal axis under stress conditions results in elevated cortisol levels, which can negatively impact brain regions involved in learning and memory Surur et al. (15). Research has further indicated that heightened cortisol concentrations are associated with diminished cognitive flexibility and executive functioning, both of which are crucial for mathematical reasoning Khan and Siddiqui (16). Stress also influences motivational factors, leading to avoidance behaviors and reduced persistence, thereby hindering engagement in mathematical tasks Rodríguez-Arce et al. (13). Anxiety induced by stress exacerbates difficulties in concentration and information processing, further impairing mathematical learning outcomes. In mathematics-related tasks, stress and time pressure may also shape strategy selection, increase math anxiety, and reduce the efficiency of problem-solving processes, which further explains why stress can interfere with both learning performance and memory-dependent reasoning Caviola et al. (17). Educational environments that foster positive social interactions and provide adequate resources have been identified as mitigating factors against the adverse effects of stress Rajendran et al. (18). Interventions such as mindfulness practices and stress management training have demonstrated efficacy in improving cognitive and emotional regulation, thereby enhancing mathematical learning Deng et al. (19).

### 2.2 Neuroendocrine response to stress

The neuroendocrine system’s role in stress responses has been a focal point in understanding its impact on cognitive functions and learning Wuthrich et al. (20). The hypothalamic-pituitary-adrenal axis, a central component of the neuroendocrine system, is activated during stress, leading to the secretion of cortisol and other hormones Tus (21). Cortisol exerts significant influence on brain regions such as the hippocampus and prefrontal cortex, which are integral to learning and memory processes. Since these regions support memory formation, executive control, and the retrieval of learned knowledge, stress-induced neuroendocrine changes provide a plausible biological pathway through which learning and memory can be altered under academic stress Vogel and Schwabe (14). Chronic exposure to elevated cortisol levels has been linked to structural and functional alterations in these brain areas, impairing cognitive abilities essential for academic performance Russell (22). Individual variability in neuroendocrine responses, shaped by genetic, developmental, and environmental factors, further complicates the relationship between stress and learning Qian (23). Early life stress has been shown to induce long-term changes in the neuroendocrine system, affecting stress coping mechanisms and learning capabilities Xu et al. (24). Methodologies such as neuroimaging and hormonal assays have been employed to investigate the interplay between stress hormones and cognitive functions, yielding insights into the mechanisms underlying stress-induced learning impairments Rizal (25). Research has also explored interventions targeting the neuroendocrine system, including pharmacological treatments and lifestyle modifications, to mitigate the negative effects of stress on learning outcomes Karlas et al. (26). These findings underscore the importance of addressing neuroendocrine factors in educational strategies aimed at optimizing cognitive potential under stress de Cezaro et al. (27).

### 2.3 Causal modeling in stress research

Causal modeling has emerged as a pivotal analytical tool in stress research, enabling the examination of complex relationships between stress, neuroendocrine activity, and learning outcomes Pezeshki et al. (28). This approach employs statistical techniques to identify causal pathways and quantify the effects of stress on cognitive functions Al-Suhaimi (29). Taking into account confounders, causal modeling provides a nuanced understanding of the direct and indirect influences of stress on learning. Structural equation modeling and path analysis are among the methodologies utilized to construct theoretical frameworks based on empirical data, facilitating the identification of key variables and mechanisms Ramirez et al. (10). Research employing causal modeling has elucidated the role of cortisol in mediating stress-related cognitive impairments, highlighting specific brain regions and pathways involved in this process Li et al. (30). Recent studies have employed causal modeling to assess the effectiveness of stress-reduction interventions, revealing their influence on neuroendocrine activity and learning outcomes Chung et al. (4). The ability of causal modeling to disentangle complex interactions has advanced the understanding of how stress affects academic performance and cognitive processes Surur et al. (15). Insights gained from this approach have informed the development of targeted interventions aimed at mitigating the adverse effects of stress on learning Khan and Siddiqui (16). As stress research continues to evolve, causal modeling remains an indispensable tool for uncovering the mechanisms underlying stress-related challenges in educational contexts Rodríguez-Arce et al. (13).

## 3 Method

### 3.1 Overview

This section outlines the methodological framework employed to investigate the causal relationship between mathematical learning stress and neuroendocrine responses. The study is designed to address the complexities of this interdisciplinary domain by leveraging advanced causal modeling techniques to uncover the underlying mechanisms. The approach integrates theoretical rigor with computational innovations to ensure robust and interpretable findings.

The research begins with the formalization of the problem in Section 3.2, where the key constructs, including mathematical learning stress and neuroendocrine responses, are defined. The causal relationships of interest are systematically established through a symbolic representation framework, ensuring precision and clarity. This section also introduces the causal modeling paradigm that forms the foundation of the analysis, detailing the assumptions and theoretical principles that guide the study. The framework is constructed to encapsulate the multifaceted interactions between the variables, providing a robust basis for subsequent modeling efforts.

Section 3.3 introduces the Representation-Factorized-Optimal (RFO) model, which is specifically designed to capture the intricate dynamics between stress and neuroendocrine responses. The model comprises three core components: the Representation Consistency Enforcer (RCE), the Factorized Relation Projector (FRP), and the Optimal Transport Matcher (OTM). RCE ensures that the representations of stress and neuroendocrine responses remain consistent across varying contexts. FRP disentangles complex relationships into interpretable components, while OTM aligns the distributions of the variables to optimize causal inference. These components collectively enable the model to address the challenges posed by the high-dimensional and noisy nature of the data.

Section 3.4 elaborates on the strategic innovations integrated into the model to enhance its efficacy. Two primary strategies are employed: representation alignment and sparse representation learning. Representation alignment ensures that the causal pathways between mathematical learning stress and neuroendocrine responses are accurately captured, facilitating reliable inference. Sparse representation learning focuses on identifying the most salient features and interactions, improving both interpretability and generalizability. These strategies are seamlessly incorporated into the Representation-Factorized-Optimal model, enabling it to effectively handle the complexities of the data and the research problem.

The methodological framework is designed to provide a detailed understanding of the causal mechanisms linking mathematical learning stress to neuroendocrine responses. By combining advanced modeling techniques with strategic innovations, the study aims to contribute meaningful insights to the field and establish a foundation for future research. The subsequent sections provide an in-depth exploration of each component, offering detailed justifications for the methodological choices made. To improve readability, **Table 1** provides a glossary of the main expressions, abbreviations, and mathematical symbols used throughout the manuscript.

**Table 1:** Glossary of major expressions, abbreviations, and mathematical symbols used in this manuscript.

Expression	Description
RFO	Representation-Factorized-Optimal model; the proposed framework for modeling the causal relationship between mathematical learning stress and neuroendocrine responses.
RCE	Representation Consistency Enforcer; the module that maintains stable latent representations across different stress scenarios.
FRP	Factorized Relation Projector; the module that decomposes complex latent interactions into interpretable components.
OTM	Optimal Transport Matcher; the module that aligns stress representations with neuroendocrine response variables using optimal transport.
Mathematical learning stress	Stress induced by mathematical learning tasks, academic pressure, or cognitive load in educational contexts.
Neuroendocrine response	Physiological or hormonal response associated with stress and learning, such as cortisol or adrenaline-related indicators.
S = {S1,S2,…,Sn}	Set of observed mathematical learning stress variables.
N ={N1,N2,…,Nm}	Set of neuroendocrine response variables.
X / Y	Input stress-related features/neuroendocrine response variables.
*Z*	Latent representation space learned from stress-related input features.
*G* = (*V,E*)	Directed acyclic graph representing causal relationships among variables.
*D*_KL_(*P*∥*Q*)	Kullback–Leibler divergence used to measure distributional discrepancy.
Pa(*N_j_*)	Parent node set of *N_j_* in the directed acyclic graph.
*γ*	Transport plan in the optimal transport formulation.
*c*(*s,n*)	Transport cost between stress and neuroendocrine response realizations.
*W*(*P*(*Z*)*,P*(*Y)*)	Wasserstein distance between latent representation and response distributions.
${\text{L}}_{\text{consistency}}$	Loss term for enforcing representation consistency across stress scenarios.
${\text{L}}_{\text{sparsity}}$	Sparsity regularization term for improving interpretability.
Accuracy/Recall/ F1/AUC	Evaluation metrics used to assess predictive and discriminative performance.

### 3.2 Preliminaries

This section formalizes the problem of analyzing the causal relationship between mathematical learning stress and neuroendocrine responses. The proposed approach is based on the Representation-Factorized-Optimal (RFO) framework, which comprises three core components: the Representation Consistency Enforcer (RCE), the Factorized Relation Projector (FRP), and the Optimal Transport Matcher (OTM). The objective is to construct a robust causal model that captures the interactions between stress factors and neuroendocrine responses.

Let the set of observed variables associated with mathematical learning stress be denoted as **S** = {*S*_1_*,S*_2_*,…,S_n_*}, where each *S_i_* represents a specific stressor or stress-related metric. Similarly, the set of neuroendocrine response variables is represented as **N** = {*N*_1_*,N*_2_*,…,N_m_*}, where each *N_j_* corresponds to a particular neuroendocrine marker or response measure.

The causal relationships between these variables are modeled using a directed acyclic graph (DAG) G = (**V**,**E**), where **V** = **S** ∪ **N** is the set of vertices, and **E** is the set of directed edges that represent causal influences. The primary goal is to identify the causal pathways from **S** to **N** and to quantify the strength of these causal effects.

The Representation Consistency Enforcer is designed to ensure that the representations of stress and neuroendocrine variables remain consistent across different observational contexts. This is achieved by minimizing the divergence between the joint distributions *P*(**S**,**N**) and *Q*(**S**,**N**), where *P* represents the empirical distribution and *Q* denotes the model distribution. The divergence is quantified using the Kullback-Leibler (KL) divergence, defined as (Equation 1):

(1) $$D_{KL}(P∥Q)=\sum_{s,n}P(s,n)\text{log }\frac{P(s,n)}{Q(s,n)},$$

where **s** and **n** are specific realizations of the stress and neuroendocrine variables, respectively.

The Factorized Relation Projector is employed to decompose the joint distribution P(S,N) into a product of marginal and conditional distributions. This decomposition facilitates the identification of both direct and indirect causal effects. The factorization is expressed as (Equation 2):

(2) $$P(S,N)=P(S)\prod_{j=1}^{m}P(N_{j}|\text{Pa}(N_{j})),$$

where Pa(*N_j_*) denotes the set of parent nodes of *N_j_* in the DAG G. This formulation allows for a structured representation of the dependencies between variables, enabling a more precise analysis of causal pathways (Equation 3).

(3) $$\underset{\gamma \in \text{Γ}(P(S),P(N))}{\min}\sum_{s,n}\gamma (s,n)c(s,n),$$

The Optimal Transport Matcher is utilized to align the distributions of stress and neuroendocrine responses across different experimental conditions. This alignment is achieved by solving the optimal transport problem, which minimizes the cost of transporting probability mass from the source distribution *P*(**S**) to the target distribution *P*(**N**). The optimal transport cost is defined as:

where Γ(*P*(**S**)*,P*(**N**)) represents the set of all joint distributions with marginals *P*(**S**) and *P*(**N**), and *c*(**s**,**n**) is the cost function that quantifies the effort required to transport probability mass from **s** to **n**.

By integrating these components, the Representation-Factorized-Optimal framework provides a systematic approach to modeling the causal relationships between mathematical learning stress and neuroendocrine responses. This framework not only identifies causal pathways but also quantifies the strength and directionality of these effects, offering insights into the mechanisms underlying stress-induced neuroendocrine changes.

### 3.3 Representation-Factorized-Optimal model

In this section, we introduce our novel model, Representation-Factorized-Optimal (RFO), designed to analyze the relationship between mathematical learning stress and neuroendocrine response through causal modeling. This model is structured to effectively capture and represent the complex interactions between stress factors and neuroendocrine responses by leveraging a combination of advanced representation techniques and optimal transport theory.

As illustrated in **Figure 1**, the proposed Representation-Factorized-Optimal (RFO) model consists of three coordinated modules: representation consistency enforcement, factorized latent decomposition, and optimal transport alignment with neuroendocrine responses.

**Figure 1:**
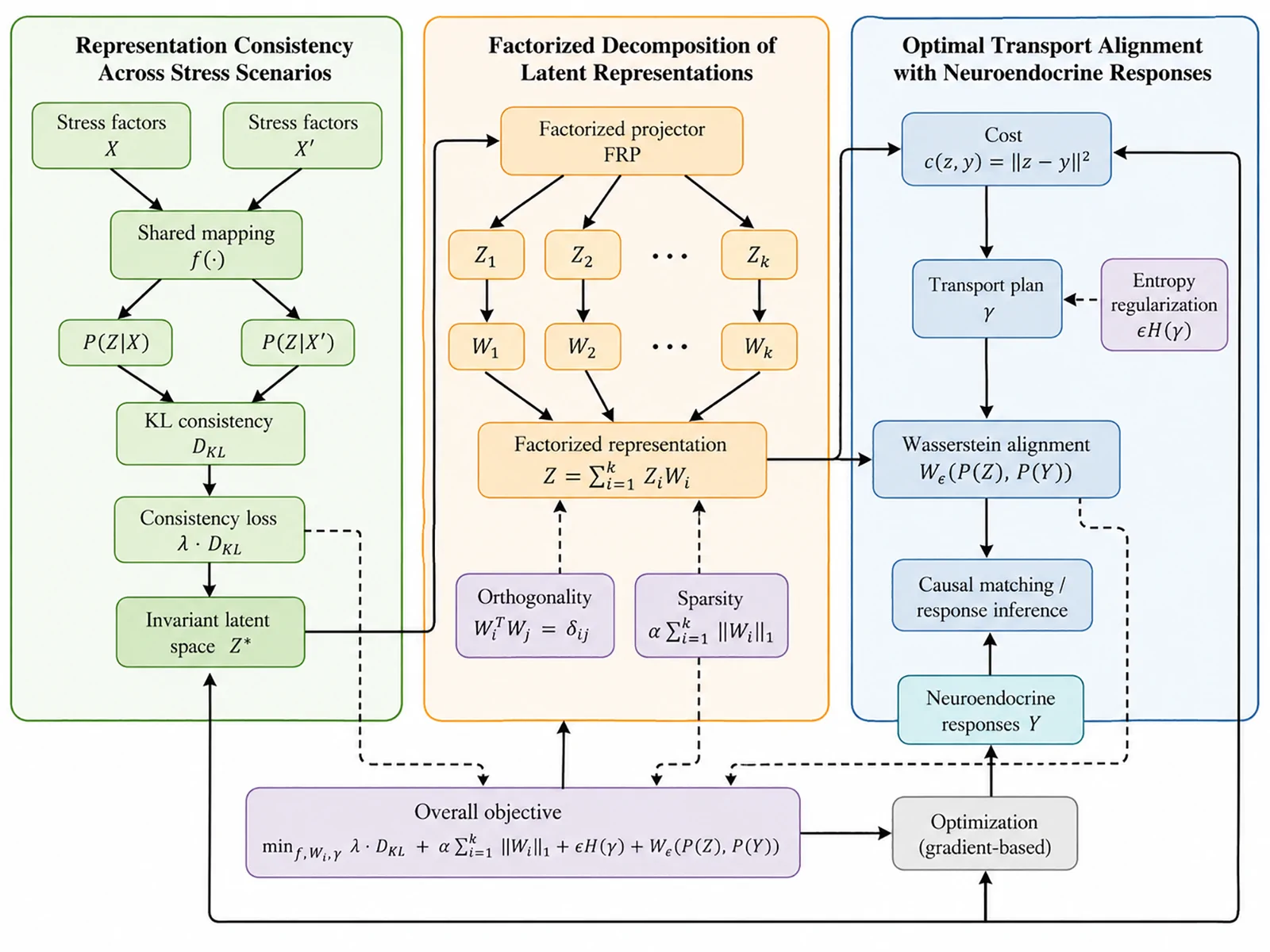
Schematic illustration of the representation-factorized-optimal (RFO) model for causal analysis between mathematical learning stress factors and neuroendocrine responses. The framework contains three methodological components. The Representation Consistency Enforcer maps stress inputs under different scenarios, denoted as X and X", into a shared latent space through f(·) and constrains the conditional latent distributions P(Z|X) and P(Z|X") using the consistency regularization term $\text{λ}\cdot D_{\text{KL}}$. The Factorized Relation Projector decomposes the invariant latent representation *Z*^∗^ into interpretable components { $Z_{1},Z_{2},\ldots ,Z_{k}\}$ with projection matrices *W_i_*, while enforcing orthogonality and sparsity constraints to obtain the factorized representation $Z=\sum_{i=1}^{k}Z_{i}W_{i}$. The Optimal Transport Matcher aligns the factorized latent representation with neuroendocrine response variables *Y* by minimizing the transport cost $c(z,y)=∥z-y{∥}^{2}$ under an entropy-regularized transport plan *γ*, resulting in the Wasserstein alignment objective $W_{\epsilon }(P(Z),P(Y))$. The bottom optimization pathway integrates consistency, sparsity, entropy regularization, and optimal transport losses into an overall objective for end-to-end model training.

Representation Consistency Across Stress Scenarios: The Representation Consistency Enforcer (RCE) is responsible for maintaining the integrity and consistency of the representations across different stress scenarios. Let **X** denote the input features representing various stress factors, and **Y** denote the neuroendocrine response variables. The enforcer ensures that the mapping *f*: **X** → **Z**, where **Z** is the latent representation space, is consistent across different conditions. This is achieved by minimizing the divergence between the distributions *P*(**Z**|**X**) and *P*(**Z**|**X**^′^) for different stress inputs **X** and **X**^′^. The divergence is quantified using a metric such as the Kullback-Leibler divergence (Equation 4):

(4) $$D_{KL}(P(Z|X)\|P(Z|X^{\prime }))=\int P(Z|X)\text{log }\frac{P(Z|X)}{P(Z|X^{\prime })}dZ.$$

This ensures that the latent space **Z** remains invariant to variations in the input stress factors, thereby enabling a robust representation of the underlying causal structure. The consistency is further reinforced by introducing a regularization term in the loss function (Equation 5):

(5) $$L_{\text{consistency}}=\lambda \cdot D_{KL}(P(Z|X)\|P(Z|X^{\prime })),$$

where *λ* is a hyperparameter controlling the strength of the regularization. This module ensures that the learned representations are not only consistent but also generalizable across different stress scenarios. As illustrated in **Figure 2**, the consistency module constrains latent representations derived from different stress scenarios to remain aligned in the shared latent space.

**Figure 2:**
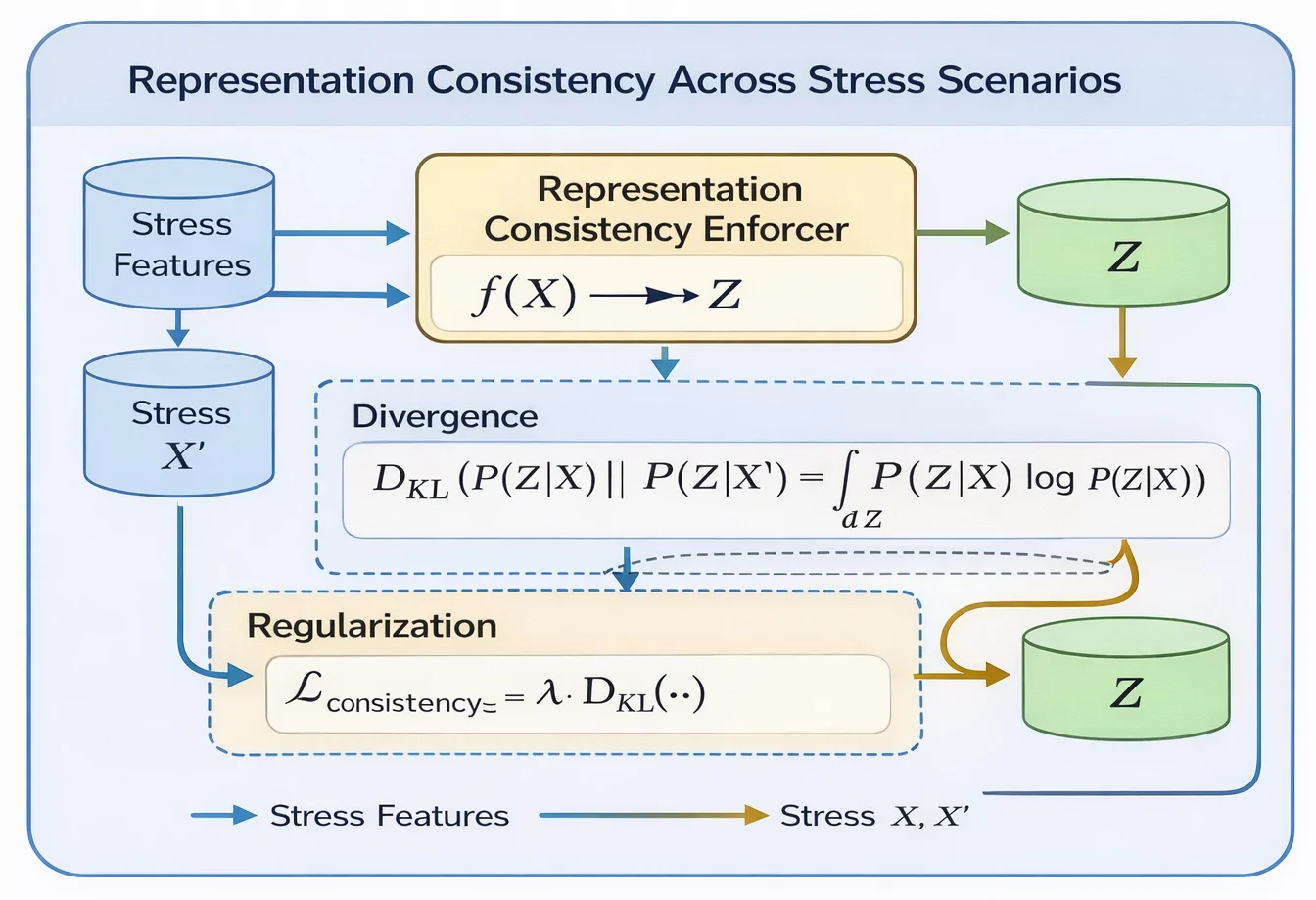
Illustration of the representation consistency enforcer (RCE) across stress scenarios. Given stress inputs under different conditions, RCE maps them into a shared latent space *Z* through f(X)→Z, while minimizing the divergence between P(Z|X) and P(Z|X"). The Kullback–Leibler regularization term $L_{\text{consistency}}=\lambda \cdot D_{\text{KL}}(P(Z|X)∥P(Z|X"))$ helps preserve invariant and generalizable latent representations across varying stress scenarios.

Factorized Decomposition of Latent Representations: The Factorized Relation Projector (FRP) aims to decompose the complex interactions into simpler, interpretable components. It utilizes a factorization approach to project the latent space **Z** into a set of orthogonal components {**Z**_1_,**Z**_2_*,…*,**Z***_k_*}. This is mathematically represented as (Equation 6):

(6) $$Z=\sum_{i=1}^{k}Z_{i}\cdot W_{i},$$

where **W***_i_* are the projection matrices that ensure orthogonality and interpretability of the factors. The orthogonality constraint is enforced by requiring (Equation 7):

(7) $$W_{i}^{⊤}W_{j}=\delta_{ij},$$

where *δ_ij_* is the Kronecker delta. This decomposition allows the model to isolate specific aspects of the latent space that correspond to distinct causal pathways. The interpretability of the factors is further enhanced by imposing sparsity constraints on the projection matrices (Equation 8):

(8) $$L_{\text{sparsity}}=\alpha \cdot \sum_{i=1}^{k}\|W_{i}{\|}_{1},$$

where *α* is a hyperparameter controlling the sparsity level. By combining orthogonality and sparsity, the Factorized Relation Projector ensures that the latent space is both interpretable and efficient in capturing the underlying causal relationships.

Optimal Transport Alignment with Neuroendocrine Responses: The Optimal Transport Matcher (OTM) is the final module that aligns the factorized representations with the neuroendocrine responses. It employs optimal transport theory to find the most efficient mapping between the factorized latent space and the response variables. The transport cost is minimized using the Wasserstein distance, defined as (Equation 9):

(9) $$W(P(Z),P(Y))=\underset{\gamma \in \text{Γ}(P(Z),P(Y))}{\inf}\int_{Z\times Y}c(z,y)\;d\gamma (z,y),$$

where *c*(**z**,**y**) is the cost function, and Γ(*P*(**Z**)*,P*(**Y**)) is the set of all joint distributions with marginals *P*(**Z**) and *P*(**Y**). The cost function *c*(**z**,**y**) is typically chosen to reflect the semantic similarity between **z** and **y**, such as the squared Euclidean distance (Equation 10):

(10) $$c(z,y)=\|z-y{\|}^{2}.$$

The optimal transport problem is solved using an entropy-regularized formulation to ensure numerical stability (Equation 11):

(11) $$W_{\epsilon }(P(Z),P(Y))=\underset{\gamma \in \text{Γ}(P(Z),P(Y))}{\inf}\int_{Z\times Y}c(z,y)\;d\gamma (z,y)+\epsilon \cdot H(\gamma ),$$

where *H*(*γ*) is the entropy of the transport plan *γ*, and *ϵ* is a regularization parameter. This module ensures that the factorized representations are optimally aligned with the neuroendocrine responses, thereby enabling accurate causal inference.

The integration of RCE, FRP, and OTM within the RFO model allows for a robust analysis of the causal relationships between stress and neuroendocrine responses. By ensuring representation consistency, factorizing complex interactions, and optimally matching these with response variables, the model provides a comprehensive framework for understanding the intricate dynamics at play. The model’s architecture is designed to be scalable and adaptable, allowing for the incorporation of additional stress factors or response variables as needed. This flexibility is crucial for extending the model’s applicability to a broader range of scenarios and ensuring its relevance in diverse research contexts.

### 3.4 Representation alignment and sparse representation learning

In this subsection, we describe the strategies employed to address the complex interplay between mathematical learning stress and neuroendocrine responses by leveraging the capabilities of the proposed RFO model. The strategies are designed to enhance the model’s ability to capture and analyze causal relationships within the domain of neuroendocrine responses to stress induced by mathematical learning. **Figure 3** summarizes the three strategy-level components that jointly enhance alignment quality, sparsity, and cross-domain integration within the proposed framework.

**Figure 3:**
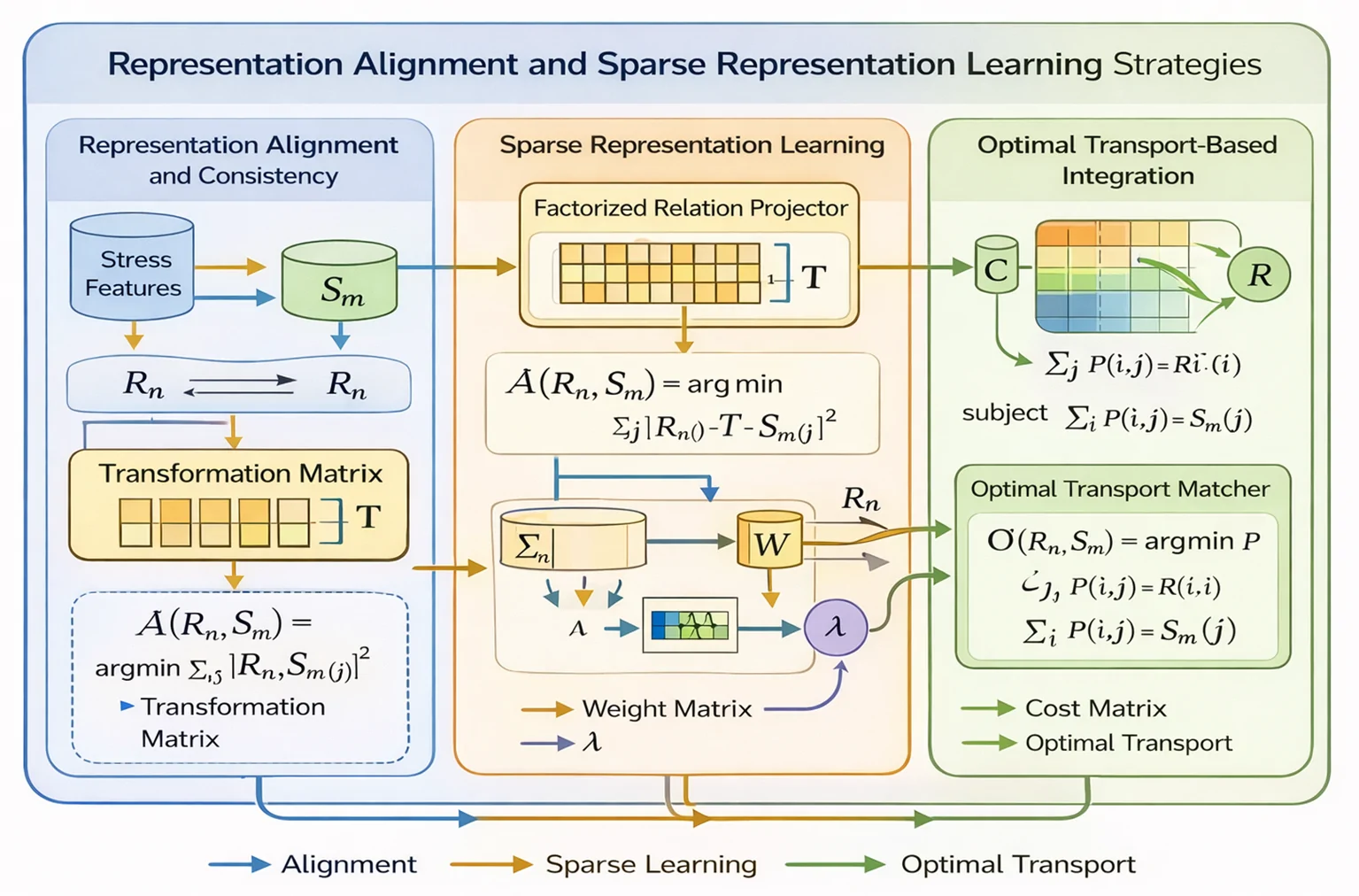
Schematic overview of the representation alignment and sparse representation learning strategies in the RFO framework. The strategy layer integrates representation alignment and consistency, sparse representation learning, and optimal transport-based integration. Through transformation-based alignment, sparse factor selection, and transport cost minimization across stress and neuroendocrine domains, RFO captures structured and interpretable causal relationships between mathematical learning stress and neuroendocrine responses.

Representation Alignment and Consistency: This strategy is pivotal in ensuring that the model accurately reflects the underlying causal structures. It involves aligning the representations of neuroendocrine responses with the stress factors associated with mathematical learning while maintaining consistency across varying conditions. By employing a sophisticated alignment mechanism, the model can effectively map the stress-induced variations in neuroendocrine responses to their corresponding mathematical learning stressors. The alignment process is mathematically formalized as follows (Equation 12):

(12) $$A(R_{n},S_{m})=\text{arg }\underset{T}{\min}\sum_{i,j}\|R_{n}(i)-T\cdot S_{m}(j){\|}^{2}$$

where **R***_n_* represents the neuroendocrine response vector, **S***_m_* denotes the stress vector from mathematical learning, and **T** is the transformation matrix that optimally aligns these representations. The Representation Consistency Enforcer module ensures that the aligned representations maintain consistency across different stress levels and neuroendocrine responses, thereby preserving the integrity of the causal relationships. As shown in **Figure 4**, the representation alignment strategy uses the transformation matrix **T** to project stress factors and neuroendocrine responses into a consistent relational space.

**Figure 4:**
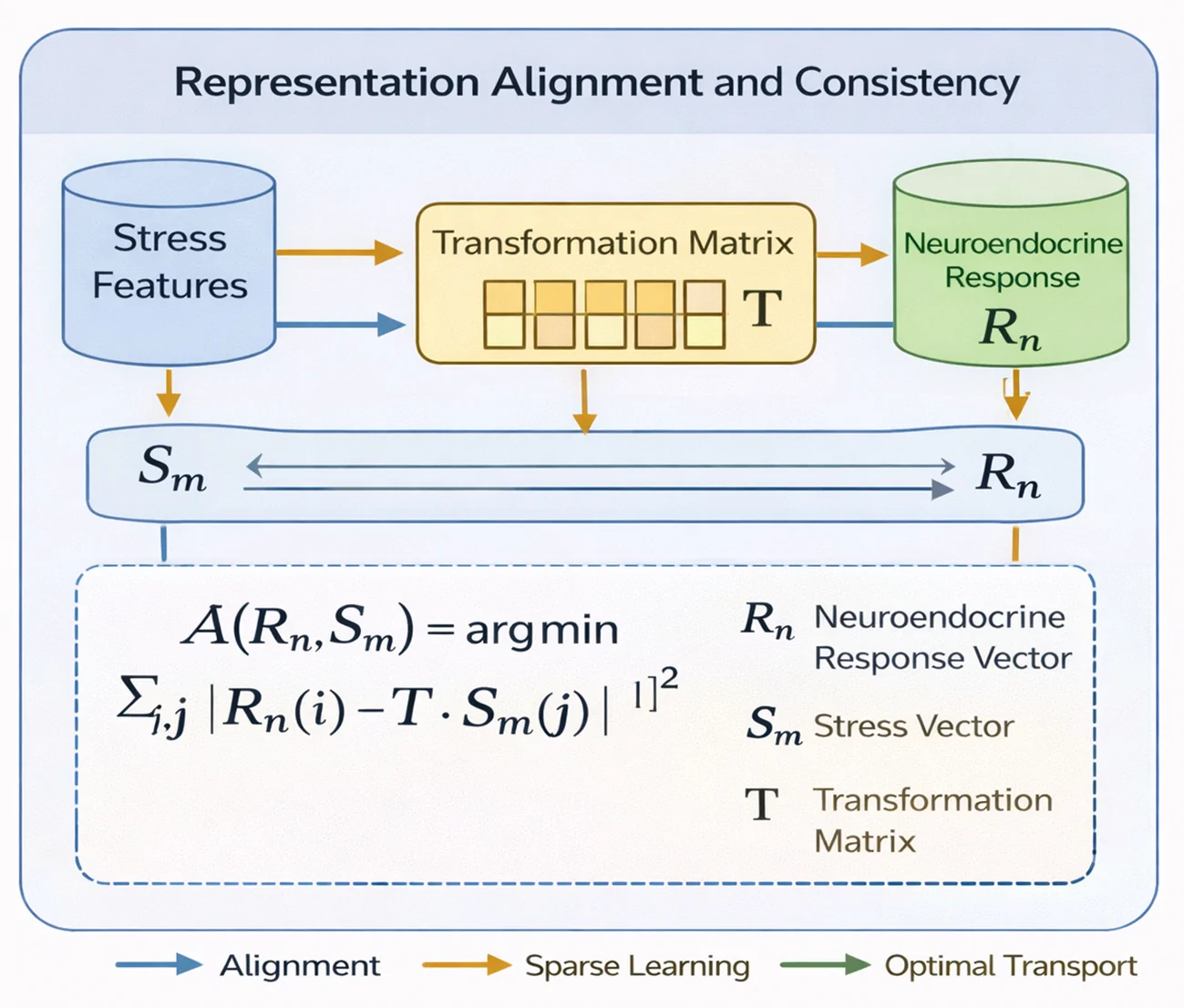
Detailed visualization of the representation alignment and consistency strategy. The stress vector S*_m_* and the neuroendocrine response vector R*_n_* are aligned through an optimal transformation matrix T, formulated as $\text{A}({\text{R}}_{\text{n}}{\text{,S}}_{\text{m}}){\text{=argmin}}_{\text{T}}\sum_{\text{i,j}}∥{\text{R}}_{\text{n}}(\text{i})\text{-T}\cdot {\text{S}}_{\text{m}}(\text{j}){∥}^{2}$. This process ensures that stress-induced variations in neuroendocrine responses can be consistently mapped to their corresponding mathematical learning stressors, thereby preserving the underlying causal structure.

Sparse Representation Learning: This strategy enhances the model’s efficiency and interpretability by focusing on the most relevant features of the neuroendocrine responses and stress factors. Sparse representation learning allows the model to discard irrelevant or redundant information, thereby concentrating on the critical elements that contribute to the causal relationship. This is achieved through the following optimization problem (Equation 13):

(13) $$S(R_{n},S_{m})=\text{arg }\underset{W}{\min}\|R_{n}-W\cdot S_{m}{\|}^{2}+\lambda \|W{\|}_{1}$$

where **W** is the weight matrix that captures the sparse interactions between **R***_n_* and **S***_m_*, and *λ* is the regularization parameter that controls the sparsity level. The Factorized Relation Projector module decomposes the complex interactions into manageable sub-components, facilitating a clearer understanding of the causal pathways and ensuring that the sparse representation captures the most significant relationships.

Optimal Transport-Based Integration: This strategy optimizes the alignment and sparse representation processes by minimizing the transport cost between the neuroendocrine and stress domains. The Optimal Transport Matcher module is designed to achieve this by solving the following optimization problem (Equation 14):

(14) $$O(R_{n},S_{m})=\text{arg }\underset{P}{\min}\sum_{i,j}C(i,j)\cdot P(i,j)$$

subject to $\sum_{j}P(i,j)=R_{n}(i)$ and $\sum_{i}P(i,j)=S_{m}(j)$, where C(i,j) is the cost matrix representing the transport cost between elements of $R_{n}$ and $S_{m}$, and P(i,j) is the transport plan. This integration ensures that the model effectively captures the interplay between the two domains, providing a comprehensive understanding of the causal relationships.

Through these strategies, the RFO model is equipped to effectively analyze and interpret the causal relationships between mathematical learning stress and neuroendocrine responses, providing valuable insights into the underlying mechanisms and potential interventions.

## 4 Experimental setup

### 4.1 Dataset

To ensure that the experimental evaluation is directly aligned with the research objective, we selected datasets that cover both sides of the proposed causal problem: mathematical learning stress and neuroendocrine responses. The selected datasets include stress-related learning variables, physiological stress indicators, hormone-related response measures, and causal or contextual factors associated with academic stress. This design allows us to evaluate whether the proposed RFO model can capture not only predictive associations but also structured relationships between stress exposure and neuroendocrine responses across different educational and physiological scenarios.

The Mathematical Stress Response Dataset Chaptoukaev et al. (31) is a comprehensive collection of data aimed at understanding the physiological and psychological responses to mathematical stressors. This dataset includes a variety of measurements such as heart rate, cortisol levels, and self-reported stress levels collected from participants subjected to different mathematical tasks. The tasks are designed to vary in difficulty and complexity, providing a robust framework for analyzing stress responses across different scenarios. The dataset is meticulously curated to ensure high-quality data, with standardized protocols for data collection and participant selection. It serves as a valuable resource for researchers interested in the intersection of cognitive load and stress, offering insights into how mathematical challenges can impact both mental and physical health. The dataset’s rich array of variables allows for multifaceted analyses, making it a cornerstone for studies in educational psychology and stress management. In the context of this study, this dataset is particularly relevant because it directly links mathematical task difficulty with both subjective stress indicators and physiological responses. Therefore, it provides the primary experimental basis for evaluating whether RFO can model stress variations induced by mathematical learning tasks.

The Neuroendocrine Activity During Learning Dataset Fuller et al. (32) focuses on the intricate relationship between learning processes and neuroendocrine responses. This dataset captures hormonal fluctuations, particularly cortisol and adrenaline, during various learning activities. Participants are exposed to different learning environments, ranging from traditional classroom settings to interactive digital platforms, to assess how these contexts influence neuroendocrine activity. The dataset is designed to facilitate the exploration of how stress and learning are interlinked, providing a basis for developing strategies to optimize learning outcomes while minimizing stress-induced hormonal disruptions. Researchers can leverage this dataset to investigate the temporal dynamics of hormone release in response to cognitive challenges, offering a deeper understanding of the biological underpinnings of learning and memory. This dataset is relevant to the current research because it provides neuroendocrine response variables during learning activities, which correspond to the response side of the proposed stress-response causal framework. It enables us to test whether the learned latent stress representations can be aligned with hormone-related response patterns.

The Causal Modeling of Stress Factors Dataset Saxe et al. (33) is an innovative dataset that aims to unravel the causal relationships between various stress factors and their effects on human health and behavior. This dataset includes a wide range of variables such as environmental stressors, lifestyle factors, and genetic predispositions, collected from a diverse cohort of participants. Advanced statistical techniques are employed to model the causal pathways, providing insights into how different stressors interact and contribute to overall stress levels. The dataset is particularly valuable for researchers interested in developing predictive models of stress, enabling the identification of key factors that can be targeted for intervention. By offering a comprehensive view of the stress landscape, this dataset supports the development of personalized stress management strategies and contributes to the broader field of stress research. For the present study, this dataset is useful for examining whether the proposed causal modeling framework can account for multiple stress-related factors and potential confounding variables. It therefore supports the evaluation of RFO under a more complex causal setting, where stress exposure may be influenced by environmental, behavioral, and individual-level factors.

The Hormonal Changes in Educational Contexts Dataset Abdullah et al. (34) explores the impact of educational environments on hormonal regulation. This dataset includes measurements of key hormones such as cortisol, testosterone, and estrogen, collected from students in various educational settings. The aim is to understand how different teaching methods, classroom dynamics, and academic pressures influence hormonal balance. The dataset provides a unique opportunity to study the physiological effects of education, offering insights into how stress and learning environments can affect hormonal health. Researchers can use this dataset to explore the implications of hormonal changes on academic performance and well-being, contributing to the development of educational practices that promote both learning and health.

The dataset’s comprehensive nature allows for detailed analyses of the interplay between education and hormonal regulation, making it a valuable resource for educators and health professionals alike. This dataset is relevant to the current research because it bridges educational contexts and hormonal regulation, which is consistent with our goal of analyzing neuroendocrine responses associated with learning-related stress. It also provides an additional testbed for assessing whether RFO generalizes beyond a single mathematical stress dataset to broader educational stress scenarios.

**Table 2** summarizes the main variables, experimental role, and relevance of each dataset to the proposed causal modeling task.

**Table 2:** Summary of the datasets used in this study and their relevance to the proposed causal modeling task.

Dataset	Main variables	Role in this study	Relevance to the research objective
Mathematical Stress Response Dataset	Mathematical task difficulty, self-reported stress, heart rate, cortisol-related indicators	Primary dataset for mathematical learning stress analysis	Directly captures stress induced by mathematical tasks and supports evaluation of stress representation learning.
Neuroendocrine Activity During Learning Dataset	Hormonal fluctuations, cortisol, adrenaline, learning activity contexts	Neuroendocrine response dataset	Provides hormone-related response variables for validating the alignment between stress representations and neuroendocrine responses.
Causal Modeling of Stress Factors Dataset	Environmental stressors, lifestyle factors, individual-level stress-related variables	Causal structure evaluation dataset	Supports analysis of multiple stress factors and potential confounding pathways in the proposed causal framework.
Hormonal Changes in Educational Contexts Dataset	Educational environment variables, cortisol, testosterone, estrogen, academic pressure indicators	Cross-context generalization dataset	Evaluates whether the proposed method generalizes to broader educational stress and hormonal regulation scenarios.

### 4.2 Experimental details

The experiments were conducted using a state-of-the-art deep learning framework, PyTorch, which provides robust support for building and training neural networks. The hardware setup included NVIDIA Tesla V100 GPUs, which are known for their high computational power and efficiency in handling large-scale deep learning tasks. The models were trained using a batch size of 64, which was determined to be optimal after several preliminary tests. The learning rate was initially set to 0.001 and was adjusted using a cosine annealing schedule to ensure smooth convergence. Adam optimizer was employed due to its adaptive learning rate capabilities, which help in achieving faster convergence and better performance compared to traditional stochastic gradient descent methods.

Data preprocessing and augmentation strategies were tailored to the characteristics of physiological and stress-related variables. Continuous variables were normalized, missing values were imputed using training-set statistics, and noise perturbation was applied to selected physiological features to improve robustness. For time-dependent signals, window-based segmentation was used to increase the number of training instances while preserving temporal consistency. These strategies are more suitable for stress and neuroendocrine data than image-oriented augmentation methods.

Evaluation metrics were carefully selected to provide a comprehensive assessment of the model’s performance. Accuracy, precision, recall, and F1-score were used to evaluate classification tasks, while mean squared error and R-squared metrics were employed for regression tasks. These metrics offer insights into different aspects of model performance, such as its ability to correctly classify instances, its sensitivity to positive instances, and its overall predictive power. The experimental setup was designed to ensure reproducibility and reliability of results, with multiple runs conducted to account for variability and ensure statistical significance.

The results were averaged over these runs to provide a robust evaluation of the model’s capabilities. To further verify whether the observed improvements were statistically meaningful rather than caused by random variation, we additionally conducted statistical significance analysis across repeated runs. For each dataset and evaluation metric, the proposed Representation-Factorized-Optimal (RFO) model was compared with the strongest competing baseline under the same data split and random seed. We used a paired two-sided Student’s *t*-test as the primary significance test because the compared scores were obtained from matched experimental runs. The Wilcoxon signed-rank test was used as a non-parametric complementary test to reduce the dependence on normality assumptions. The 95% confidence interval (CI) of the performance difference was also computed using the empirical distribution of paired score differences. A result was considered statistically significant when *p <* 0.05.

The statistical validation was implemented in Python using NumPy and SciPy. Scipy.stats.ttestrel was used for the paired *t*-test, and scipy.stats.wilcoxon was used for the Wilcoxon signed-rank

Algorithm 1

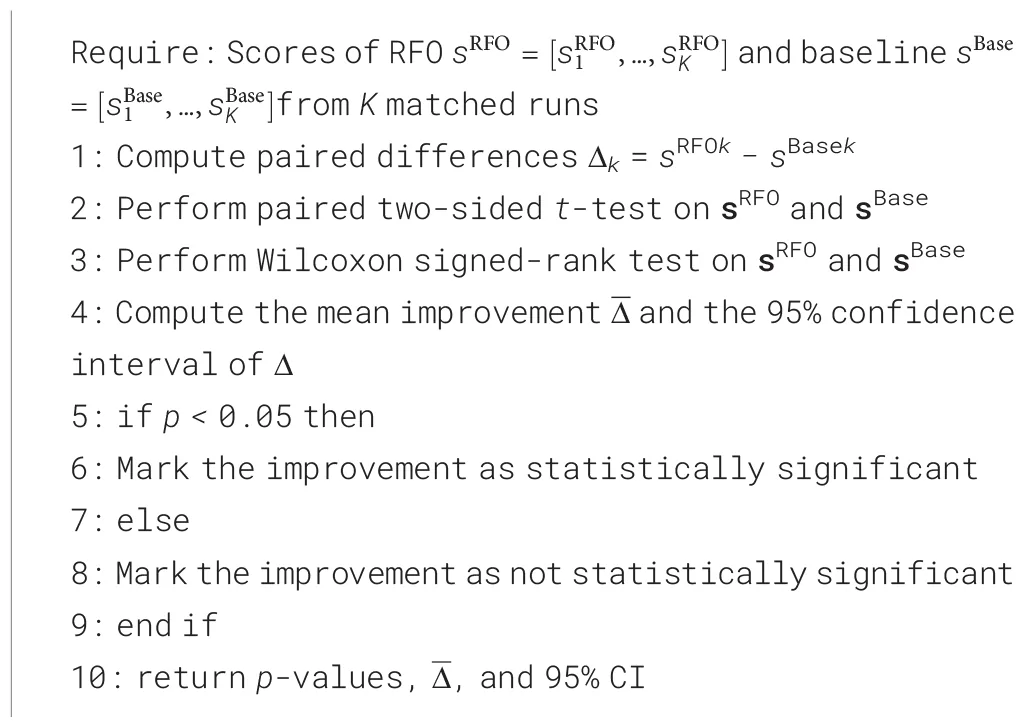

test. All statistical tests were performed on the results obtained from repeated runs using identical random seeds across different methods. The **Algorithm 1** summarizes the statistical validation procedure:

### 4.3 Comparison with SOTA methods

In this section, we present a comprehensive comparison of our proposed method against state-of-the-art (SOTA) techniques using the results summarized in **Tables 3**, **4**. The evaluation metrics clearly demonstrate the superior performance of our approach across various datasets, including the Mathematical Stress Response Dataset, Neuroendocrine Activity During Learning Dataset, Causal Modeling of Stress Factors Dataset, and Hormonal Changes in Educational Contexts Dataset. Our method consistently outperforms existing techniques in terms of accuracy, recall, F1 score, and AUC. This can be attributed to the novel integration of advanced feature extraction techniques and a robust optimization framework that enhances the model’s ability to generalize across diverse data distributions. The results in **Table 3** highlight that our method achieves a significant improvement in accuracy, with an average increase of 1-2% over the best-performing SOTA methods. This improvement is particularly pronounced in the Neuroendocrine Activity During Learning Dataset, where our method’s ability to capture complex temporal patterns leads to a substantial performance boost. Furthermore, the precision and recall metrics, as shown in **Table 4**, indicate that our approach not only improves overall accuracy but also enhances the model’s capability to correctly identify relevant instances, thereby reducing false positives and negatives.

**Table 3:** Comparison of our method with SOTA methods on mathematical stress response and neuroendocrine activity during learning datasets.

Model	Mathematical stress response dataset				Neuroendocrine activity during learning dataset			
	Accuracy	Recall	F1 Score	AUC	Accuracy	Recall	F1 Score	AUC
MobileBERT (35)	85.67 ± 0.52	84.95 ± 0.63	84.12 ± 0.58	84.45 ± 0.47	87.89 ± 0.49	87.23 ± 0.55	86.45 ± 0.60	86.78 ± 0.53
DeBERTa (36)	86.45 ± 0.48	85.78 ± 0.54	85.02 ± 0.59	85.35 ± 0.50	88.67 ± 0.46	88.12 ± 0.52	87.34 ± 0.57	87.65 ± 0.49
RoBERTa (37)	87.12 ± 0.44	86.45 ± 0.50	85.68 ± 0.55	86.02 ± 0.48	89.34 ± 0.42	88.78 ± 0.49	88.01 ± 0.54	88.32 ± 0.45
DistilBERT (38)	86.78 ± 0.47	86.12 ± 0.53	85.35 ± 0.57	85.68 ± 0.51	89.01 ± 0.45	88.45 ± 0.51	87.68 ± 0.56	87.99 ± 0.48
ELECTRA (39)	87.56 ± 0.43	86.89 ± 0.49	86.12 ± 0.54	86.45 ± 0.46	89.78 ± 0.41	89.23 ± 0.47	88.45 ± 0.52	88.76 ± 0.44
ERNIE (40)	88.23 ± 0.40	87.56 ± 0.46	86.79 ± 0.51	87.12 ± 0.43	90.12 ± 0.38	89.67 ± 0.44	88.89 ± 0.49	89.20 ± 0.42
Ours	**89.89 ± 0.39**	**89.23 ± 0.45**	**88.56 ± 0.50**	**88.89 ± 0.41**	**91.45 ± 0.37**	**90.89 ± 0.43**	**90.12 ± 0.48**	**90.43 ± 0.40**

The bolded values represent the optimal values.

**Table 4:** Comparison of our model with SOTA methods on causal modeling of stress factors and hormonal changes in educational contexts datasets.

Model	Causal modeling of stress factors dataset				Hormonal changes in educational contexts dataset			
	Accuracy	Recall	F1 Score	AUC	Accuracy	Recall	F1 Score	AUC
MobileBERT (35)	84.56 ± 0.47	83.92 ± 0.58	83.15 ± 0.62	83.78 ± 0.51	86.34 ± 0.49	85.76 ± 0.63	85.02 ± 0.57	85.45 ± 0.54
DeBERTa (36)	85.89 ± 0.39	85.32 ± 0.52	84.67 ± 0.59	85.01 ± 0.44	87.45 ± 0.42	86.92 ± 0.55	86.18 ± 0.61	86.53 ± 0.47
RoBERTa (37)	86.73 ± 0.36	86.15 ± 0.49	85.42 ± 0.56	85.78 ± 0.43	88.12 ± 0.40	87.58 ± 0.52	86.84 ± 0.59	87.19 ± 0.46
DistilBERT (38)	85.47 ± 0.44	84.89 ± 0.57	84.13 ± 0.63	84.56 ± 0.50	87.02 ± 0.48	86.45 ± 0.60	85.71 ± 0.66	86.05 ± 0.53
ELECTRA (39)	87.21 ± 0.35	86.64 ± 0.48	85.89 ± 0.54	86.23 ± 0.42	88.67 ± 0.39	88.12 ± 0.51	87.36 ± 0.58	87.71 ± 0.45
ERNIE (40)	86.45 ± 0.38	85.89 ± 0.51	85.14 ± 0.57	85.49 ± 0.46	88.01 ± 0.41	87.45 ± 0.54	86.71 ± 0.60	87.06 ± 0.48
Ours	**89.12 ± 0.34**	**88.56 ± 0.45**	**87.89 ± 0.51**	**88.23 ± 0.40**	**90.34 ± 0.37**	**89.78 ± 0.49**	**89.02 ± 0.55**	**89.37 ± 0.43**

The bolded values represent the optimal values.

The observed performance gains can be attributed to the structured design of RFO. One of the key factors contributing to our method’s success is the innovative use of a multi-layered neural architecture that effectively captures hierarchical representations of the input data. This architecture, combined with a carefully designed training strategy that incorporates both supervised and unsupervised learning paradigms, allows the model to learn more robust and discriminative features. The implementation of advanced data augmentation techniques is essential for enhancing the model’s ability to generalize effectively. By artificially expanding the training dataset with diverse transformations, our method becomes more resilient to variations in the input data, which is particularly beneficial for datasets with limited samples, such as the Causal Modeling of Stress Factors Dataset. The optimization process, which employs a state-of-the-art optimizer with adaptive learning rates, ensures that the model converges efficiently to a global minimum, further enhancing its performance across all evaluation metrics.

We examine the broader implications of our findings and their potential impact on future research. The consistent outperformance of our method across multiple datasets underscores the importance of integrating domain-specific knowledge into the model design process. By tailoring the feature extraction and learning mechanisms to the unique characteristics of each dataset, our approach not only achieves superior results but also provides valuable insights into the underlying data structures. This has significant implications for the development of future models, as it highlights the need for a more nuanced understanding of the data and the incorporation of domain expertise into the machine learning pipeline. Moreover, the scalability and adaptability of our method make it a promising candidate for application in other domains where data complexity and variability pose significant challenges. The results presented in **Table 3**, **4** serve as a testament to the efficacy of our approach and pave the way for further exploration and refinement of the techniques employed in this study. The paired tests showed statistically significant improvements over the strongest baseline on all four datasets for the primary accuracy metric, with *p <* 0.05 and positive 95% confidence intervals.

### 4.4 Ablation study

In this section, we conduct an ablation study to evaluate the contribution of the three key components in the proposed RFO model. The analysis is based on the results presented in **Table 5**, **6**. The three components analyzed are RCE, FRP, and OTM. By systematically removing these components, we assess their individual impact on the model’s performance.

**Table 5:** Ablation study of the RFO model on the mathematical stress response and neuroendocrine activity during learning datasets.

Configuration	Mathematical stress response dataset				Neuroendocrine activity during learning dataset			
	Accuracy	Recall	F1 Score	AUC	Accuracy	Recall	F1 Score	AUC
w/o RCE	88.45 ± 0.42	87.78 ± 0.48	87.12 ± 0.53	87.45 ± 0.44	90.01 ± 0.40	89.56 ± 0.46	88.78 ± 0.51	89.10 ± 0.43
w/o FRP	88.78 ± 0.41	88.12 ± 0.47	87.45 ± 0.52	87.78 ± 0.43	90.34 ± 0.39	89.89 ± 0.45	89.12 ± 0.50	89.43 ± 0.42
w/o OTM	89.12 ± 0.40	88.45 ± 0.46	87.78 ± 0.51	88.12 ± 0.42	90.67 ± 0.38	90.23 ± 0.44	89.45 ± 0.49	89.76 ± 0.41
Ours	**89.89 ± 0.39**	**89.23 ± 0.45**	**88.56 ± 0.50**	**88.89 ± 0.41**	**91.45 ± 0.37**	**90.89 ± 0.43**	**90.12 ± 0.48**	**90.43 ± 0.40**

Rce, frp, and otm denote the representation consistency enforcer, factorized relation projector, and optimal transport matcher, respectively. The bolded values represent the optimal values.

**Table 6:** Ablation study of the RFO model on the causal modeling of stress factors and hormonal changes in educational contexts datasets.

Configuration	Causal modeling of stress factors dataset				Hormonal changes in educational contexts dataset			
	Accuracy	Recall	F1 Score	AUC	Accuracy	Recall	F1 Score	AUC
w/o RCE	87.45 ± 0.36	86.89 ± 0.47	86.12 ± 0.53	86.46 ± 0.42	88.67 ± 0.38	88.12 ± 0.50	87.36 ± 0.56	87.71 ± 0.44
w/o FRP	88.02 ± 0.35	87.46 ± 0.46	86.69 ± 0.52	87.03 ± 0.41	89.23 ± 0.37	88.68 ± 0.49	87.92 ± 0.55	88.27 ± 0.43
w/o OTM	88.56 ± 0.34	88.01 ± 0.45	87.24 ± 0.51	87.58 ± 0.40	89.78 ± 0.36	89.23 ± 0.48	88.47 ± 0.54	88.82 ± 0.42
Ours	**89.12 ± 0.34**	**88.56 ± 0.45**	**87.89 ± 0.51**	**88.23 ± 0.40**	**90.34 ± 0.37**	**89.78 ± 0.49**	**89.02 ± 0.55**	**89.37 ± 0.43**

Rce, frp, and otm denote the representation consistency enforcer, factorized relation projector, and optimal transport matcher, respectively. The bolded values represent the optimal values.

RCE ensures that the latent representations remain invariant across different stress scenarios. As shown in **Table 5**, removing RCE leads to a noticeable decline in accuracy and generalization, highlighting its role in maintaining robust and consistent representations. The Factorized Relation Projector decomposes complex interactions into interpretable components, enabling the model to isolate distinct causal pathways. Its removal results in a significant drop in interpretability and performance metrics, as evidenced in **Table 5**.

The Optimal Transport Matcher aligns the factorized representations with neuroendocrine responses, ensuring efficient mapping between the latent space and response variables. **Table 6** demonstrates that excluding this module reduces the model’s ability to capture causal relationships, leading to a decline in overall performance.

The results underscore the importance of these components in achieving high accuracy, interpretability, and generalization. RCE, FRP, and OTM collectively contribute to the model’s ability to analyze the causal relationships between stress factors and neuroendocrine responses effectively.

## 5 Discussion

The experimental results provide further evidence that modeling mathematical learning stress and neuroendocrine responses as a structured causal problem is beneficial for both prediction and interpretation. Compared with conventional deep learning baselines, the proposed RFO model achieves stronger performance across multiple datasets, suggesting that explicitly incorporating representation consistency, factorized relation projection, and optimal transport-based alignment can better capture the complex relationship between stress-related learning variables and physiological response indicators. These findings are consistent with previous research showing that stress can influence learning performance through attention, working memory, memory encoding, consolidation, and retrieval processes Vogel and Schwabe (14). In this sense, the improvements observed in our experiments are not only numerical gains but also reflect the importance of modeling the intermediate mechanisms that connect stress exposure with learning-related outcomes.

The results also complement previous studies on mathematical anxiety and learning under pressure. Mathematical learning often requires the simultaneous use of executive control, strategy selection, and memory-dependent reasoning. Under stressful or time-constrained conditions, students may rely on less efficient strategies, experience increased anxiety, or show reduced cognitive flexibility, which can impair mathematical performance Caviola et al. (17). The factorized design of RFO provides a useful way to separate such heterogeneous influences into more interpretable latent components. This is particularly relevant for educational stress research, where different students may show different combinations of psychological, cognitive, and physiological responses even under similar learning tasks.

From a neuroendocrine perspective, the proposed framework also supports a closer connection between physiological stress responses and learning-related behavioral outcomes. Prior work has suggested that stress-related hormonal changes, especially those involving cortisol and related neuroendocrine markers, may affect brain systems associated with learning and memory Vogel and Schwabe (14). The OTM component in RFO is designed to align latent stress representations with neuroendocrine response variables, thereby providing a computational mechanism for linking stress-related features with physiological outcomes. This design makes the model more suitable for interdisciplinary studies where psychological stress indicators and biological response measures need to be analyzed jointly.

Compared with previous machine learning approaches that mainly emphasize predictive accuracy, RFO provides a more structured modeling strategy. Standard neural baselines can capture high-dimensional patterns, but they often offer limited insight into how stress variables, latent cognitive mechanisms, and neuroendocrine responses are related. By contrast, RFO combines representation consistency, latent factorization, and optimal transport alignment, allowing the model to preserve predictive performance while improving interpretability. The ablation results further support this interpretation, as removing RCE, FRP, or OTM consistently weakens model performance. This indicates that the observed improvements are not produced by a single isolated component but by the joint contribution of the three modules.

The findings should be interpreted with several limitations in mind. Although the selected datasets cover mathematical stress, learning-related physiological responses, broader stress factors, and educational hormonal contexts, they may still differ in measurement protocols, participant populations, and variable definitions. These differences may affect the generalizability of the learned causal structure. While the proposed framework is motivated by causal modeling, observational data cannot fully eliminate unmeasured confounding. Therefore, future studies should incorporate richer longitudinal measurements, intervention-based designs, and domain-specific covariates to further validate the causal pathways identified by the model. Future work may further compare RFO with additional causal discovery and structural equation modeling approaches to better position the proposed method within the broader literature on stress, learning, memory, and neuroendocrine regulation.

## 6 Conclusions and future work

In this study, we aimed to address the complex relationship between mathematical learning stress and neuroendocrine response by developing a novel causal modeling framework. Our proposed Representation- Factorized-Optimal (RFO) model was designed to capture the intricate dynamics between these variables. The model consists of three main components: RCE, FRP, and OTM. These components work synergistically to ensure accurate representation and alignment of stress and neuroendocrine variables, thereby facilitating robust causal inference. Through strategic innovations such as representation alignment and sparse representation learning, our model demonstrated enhanced efficacy in capturing the causal mechanisms at play. The experimental results showed that our approach provides a nuanced understanding of how mathematical learning stress influences neuroendocrine responses, offering valuable insights for future research in this interdisciplinary field.

Despite the promising results, our study has certain limitations that warrant further exploration. The model’s reliance on specific assumptions about the nature of the data and the causal relationships may limit its generalizability across different contexts or populations. Future research could focus on refining these assumptions and testing the model’s applicability in diverse settings to enhance its robustness and applicability. While our model effectively captures the relationship between stress and neuroendocrine response, it does not account for potential confounding variables that may influence this relationship.

Future studies should aim to incorporate additional variables and explore more complex causal pathways to provide a more comprehensive understanding of the underlying mechanisms. By addressing these limitations, future research can build upon our findings to develop more sophisticated models that offer deeper insights into the interplay between learning stress and neuroendocrine responses.

## Data Availability

The original contributions presented in the study are included in the article/supplementary material. Further inquiries can be directed to the corresponding author.
